# Insights into the role of collided ribosomes during the activation of the integrated stress response

**DOI:** 10.1042/BST20253034

**Published:** 2025-05-28

**Authors:** Ankanahalli N. Nanjaraj Urs, Lucas Kim, Hani S. Zaher

**Affiliations:** Department of Biology, Washington University in St. Louis, Campus Box 1137, One Brookings Drive, St. Louis, MO, U.S.A.

**Keywords:** collided ribosomes, Gcn2, Gcn4, ribosome quality control, the integrated stress response, translational control

## Abstract

Mechanisms that regulate and reprogram gene expression are particularly important under stress conditions. The integrated stress response (ISR) signaling pathway is one such pro-survival and adaptive mechanism conserved in eukaryotes. The ISR is characterized by the activation of protein kinases that phosphorylate the eukaryotic initiation factor 2α (eIF2α) in response to several stress conditions, including nutrient deprivation, viral infection, and protein misfolding. Phosphorylation of eIF2α results in global inhibition of translation, while promoting the translation of a few pro-survival genes. Here, we focus on the mechanism of activation of the eIF2α kinase general control nonderepressible 2 (Gcn2). The protein was initially discovered in yeast more than four decades ago, and it was proposed to respond to amino acid starvation through the accumulation of deacylated tRNAs. However, more recent studies have changed our understanding of its activation and suggest a direct role for ribosome stalling and collisions in the process. In this review, we discuss the classical model for the tRNA-mediated activation of GCN2 and the recent shift in this model to accommodate the observations that wide-ranging translational stresses trigger its activation.

## Introduction

Maintaining cellular homeostasis is a defining feature of life. To do so, organisms carefully reprogram gene expression in response to environmental changes, which becomes increasingly important under conditions of stress. Many of these adaptive mechanisms arose early in evolution and remain conserved to the present day, highlighting their importance for organismal fitness. In eukaryotes, the integrated stress response (ISR) is an important mechanism because of its ability to respond to a diverse set of biotic and abiotic stress conditions, which include nutrient deprivation, viral infection, protein misfolding, hypoxia, heat shock, and alkylative/oxidative damages to nucleobases [1,2]. The term 'integrated' of the ISR stems from the observation that all these stress conditions, although sensed through different mechanisms, converge downstream on the phosphorylation of the α subunit of eukaryotic initiation factor 2 (eIF2α) on serine 51, which has profound consequences on global protein synthesis. In particular, phosphorylation of eIF2α leads to a global reduction in translation, while at the same time promoting the translation of several pro-survival mRNAs. Under prolonged stress that causes irreparable damage, the response pathway is switched to up-regulate apoptotic responses [3–6].

Mammals have four eIF2α kinases with extensive homology in their kinase catalytic domains but distinct regulatory domains. General control nonderepressible 2 (GCN2) kinase primarily responds to amino acid starvation or translational stresses. Protein kinase R (PKR) is typically activated by viral threats to the cell, specifically through the presence of viral dsRNA. PKR-like endoplasmic reticulum kinase (PERK) is activated by the accumulation of unfolded proteins within the endoplasmic reticulum stress. Heme-regulated inhibitor (HRI) kinase is activated in response to cytoplasmic protein misfolding [1,6,7]. Each of these kinases is known to dimerize and auto-phosphorylate for their full activation. Among these, GCN2 appears to be the ancestral eIF2α kinase, as it is conserved across all eukaryotes and is the only one found in yeast [8,9].

Here, we review our current understanding of the mechanism of GCN2 activation, focusing on recent studies that highlighted a crucial role for ribosome stalling during the process. While we do not discuss the activation of the other eIF2α kinases, we briefly discuss how the phosphorylation of the initiation factor leads to translational reprogramming. Finally, we offer our perspective on the interplay between the ISR and other signaling and quality-control processes.

### Gcn2 is an eIF2α kinase

Prior to the discovery of Gcn2 in yeast, it was known that translation in rabbit reticulocyte extract is inhibited in the absence of hemin, in the presence of double-stranded RNA, and under oxidative conditions. Interestingly, regardless of the stress conditions, protein synthesis was derepressed by adding excess eIF2, suggesting that this initiation factor is the target of inhibition [10]. Later studies established that the repression of translation is mediated through phosphorylation of eIF2 by HRI and PKR, inhibiting its binding to the initiator tRNA [7,11,12].

In contrast with HRI and PKR, Gcn2 was initially identified as a positive regulator of the general amino acid control. Briefly, in yeast, deprivation of a single amino acid leads to derepression of genes involved in the synthesis of several amino acids, which requires the translation of the transcription factor *GCN4* [13]. In the absence of *GCN2*, translation of *GCN4* cannot be derepressed [14,15]. Sequence analysis of GCN2 revealed that its catalytic domain is similar to those of HRI and PKR, suggesting that the factor is similarly an eIF2α kinase, linking amino acid starvation to translational reprogramming [16]. Indeed, in 1992, Dever and colleagues showed that Gcn2 phosphorylates eIF2α at serine 51 [17]. More importantly, its kinase activity was shown to be modulated by amino acid availability and is required for the derepression of *GCN4* translation. These findings recognized eIF2α phosphorylation beyond its established role in inhibiting translation initiation and arguably launched the field of translational control of gene expression.

### Consequences of eIF2α phosphorylation

**Figure 1:**
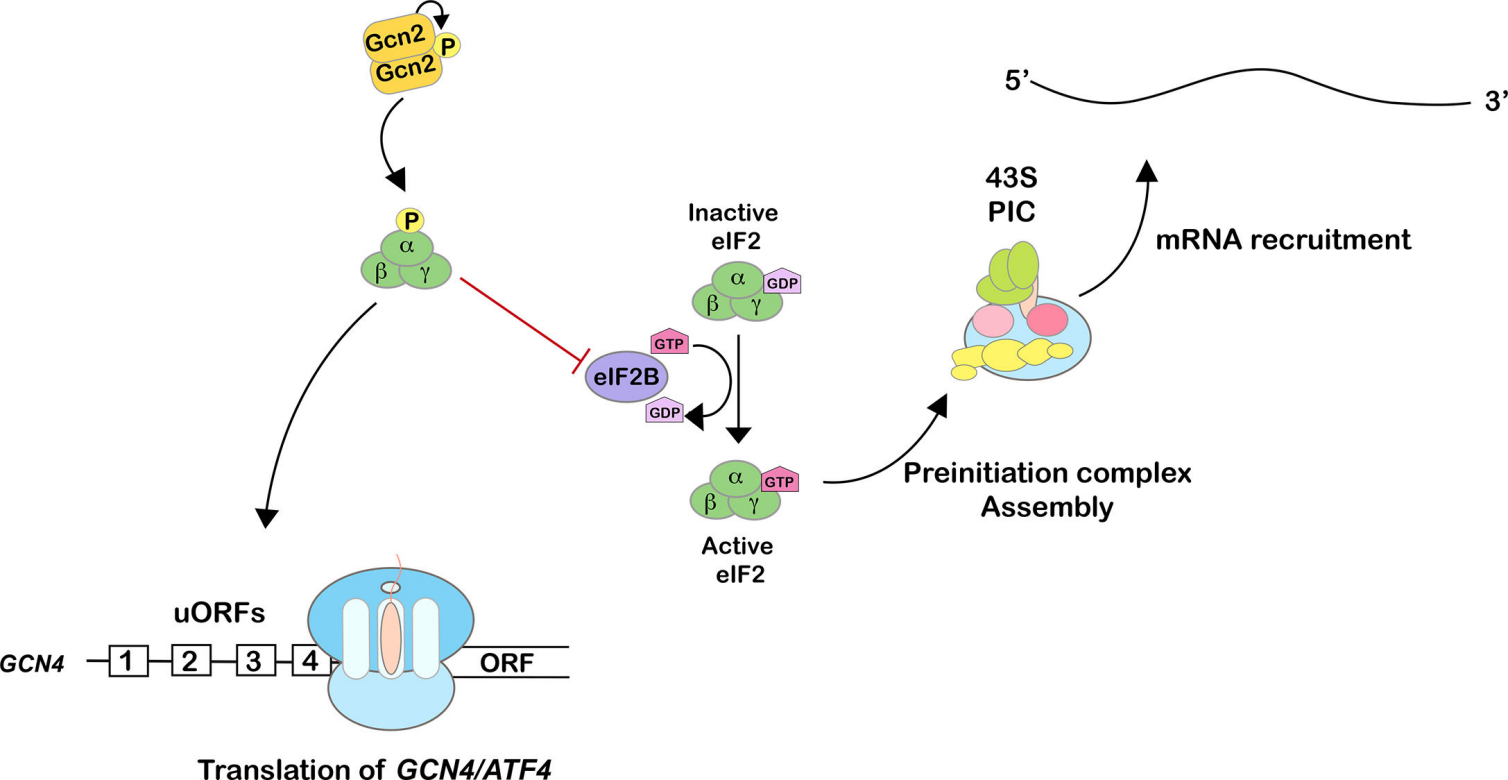
Phosphorylation of eukaryotic initiation factor 2α (eIF2α) inhibits canonical translation initiation while promoting the translation of GCN4. Gcn2-mediated phosphorylation of eIF2α inhibits the exchange activity of eIF2B, leading to a reduction in the concentration of initiator tRNA ternary complex and downstream inhibition of preinitiation complex assembly. As a result, global translation is inhibited, while that of *GCN4* is derepressed because of the bypass of the inhibitory effect of its uORFs.

During translation initiation, Met-tRNA_i_^Met^ is delivered to the small subunit in a ternary complex with eIF2 and GTP, which is hydrolyzed prior to start-codon selection [18,19]. To bind a new initiator tRNA, the factor must exchange its bound GDP for a GTP, a reaction catalyzed by the nucleotide exchange factor eIF2B. eIF2 is a heterotrimeric protein composed of α, β, and γ subunits. When serine 51 of the α subunit is phosphorylated, eIF2 binds eIF2B more tightly, effectively acting as a competitive inhibitor and preventing the exchange of GDP to GTP on eIF2 (Figure 1). This limits the availability of the ternary complex and, as a result, leads to a global reduction in protein synthesis [9,20–23]. At the same time, a reduction in the ternary complex concentration promotes the translation of a subset of mRNAs, whose encoded protein products are important to adapt to stress conditions. In particular, central to the induction of the ISR is the translation of its key effector *GCN4* in yeast and *ATF4* in mammals (Figure 1). Both are transcription factors with hundreds of targets with diverse biological roles, including amino acid biosynthesis enzymes and transporters, autophagy, peroxisome, and mitochondrial function [3,5,24–27]. As importantly, they undergo an atypical translation–initiation mechanism that is sensitive to the concentration of the ternary complex. *GCN4* and *ATF4* transcripts harbor upstream open reading frames (uORFs), which inhibit the translation of the main ORF under normal conditions [3,28]. In particular, the 5′-UTR of *GCN4* contains four such uORFs. Ribosomes initiate translation on uORF1, but ribosome recycling is inefficient on this uORF, and the small subunit often fails to dissociate post-termination and resumes scanning downstream. When the concentration of the ternary complex is relatively high, the subunit is able to bind a new complex and initiate protein synthesis on one of the downstream uORFs. Ribosome recycling on these uORFs is very efficient, and the small subunit dissociates, failing to scan to the main ORF of the gene. When the concentration of the ternary complex is relatively low, the small subunit is unable to pick up a new ternary complex in time to initiate translation on the downstream uORFs. Instead, translation initiates on the main ORF, producing the transcription factor [19,29,30].

### Early insights into Gcn2 activation

**Figure 2:**
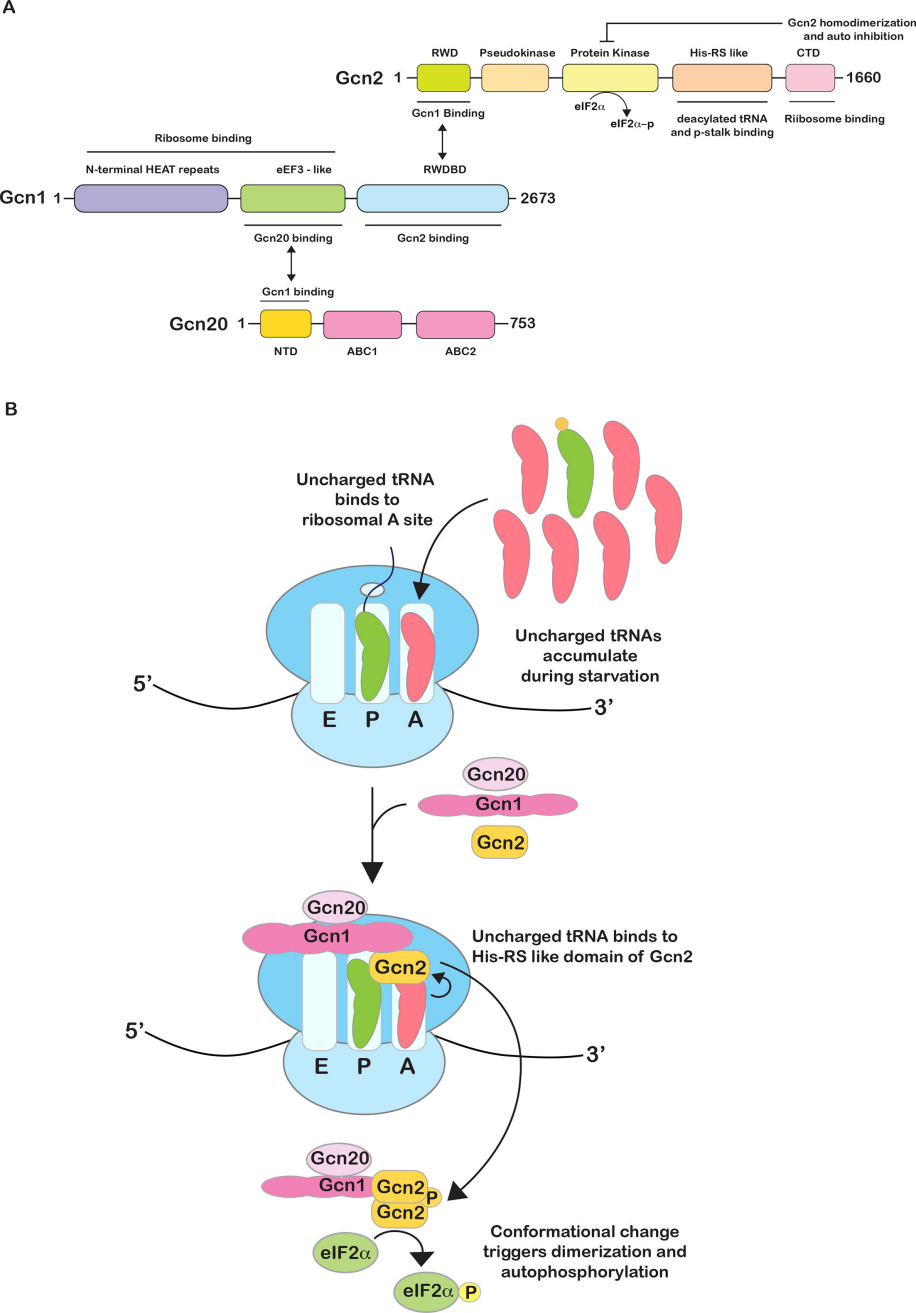
The classical model for Gcn2 activation by deacylated tRNAs. (**A**) Domain architecture of yeast Gcn1, Gcn2, and Gcn20. (**B**) Model for Gcn2 activation by deacylated tRNA. Deacylated tRNAs (shown in pink with no charged groups, as opposed to charged tRNA shown in green) compete with cognate charged tRNAs for the ribosomal A site during amino acid starvation owing to their increased accumulation. Direct physical interaction between Gcn1 and Gcn2 facilitates the binding of uncharged tRNA with the His-RS-like domain of Gcn2 on the ribosome or functions to eject uncharged tRNAs from the A site and deliver to Gcn2, triggering dimerization and autophosphorylation of the kinase domain, leading to the phosphorylation of eIF2α.

Early clues into the mechanism of Gcn2 activation came from the analysis of its domain architecture and those of its co-activators. Gcn2 is a multidomain protein with key regulatory regions, including an RWD domain, a pseudokinase domain, a protein kinase domain, a HisRS-like domain (similar to histidyl-tRNA synthetase), and a C-terminal domain (CTD) [31–36] (Figure 2A). Unlike other eIF2α kinases, which largely function on their own, Gcn2 requires the presence of at least one factor for its activity. In the absence of Gcn1, eIF2α phosphorylation is nearly completely inhibited when yeast is starved for amino acids. Gcn1 is a highly conserved large protein [37]. The protein has extensive HEAT repeats, with the central domain being highly homologous with eukaryotic elongation factor 3 (eEF3). This central domain, together with the N-terminal domain, is required to interact with the ribosome. The C-terminal RWD domain is required to mediate a direct interaction with the N-terminal RWD domain of Gcn2 [37,38] (Figure 2A). In addition to Gcn1, robust Gcn2 activation requires the presence of Gcn20, which neither interacts directly with Gcn2 nor with ribosomes. The protein is composed of an N-terminal domain (NTD) and two ATP-binding cassette domains. The NTD is required for interaction with Gcn1, and this domain is sufficient for stabilizing Gcn1 on the ribosome and Gcn2 activation (Figure 2A). Thus, the stimulatory effect of Gcn20 on Gcn2 activation is through Gcn1 [33,39,40].

The observation that Gcn2 harbors a HisRS domain, and that its co-activators bind ribosomes, evoked a model for its activation akin to that of the stringent response by RelA in bacteria. Here, amino acid starvation leads to the accumulation of deacylated tRNAs on ribosomes that bind RelA’s ThrRS, GTPase, and SpoT/RelA (TGS) domain, stimulating its ppGpp synthesis activity [41,42]. In an analogous manner, ribosome-bound deacylated tRNAs were thought to activate Gcn2 through the action of Gcn1 [34,38]. Unlike eEF3, which facilitates the removal of deacylated tRNAs from the E site, Gcn1’s eEF3 domain is thought to deliver them to Gcn2 for its activation [8,43,44] (Figure 2B). The binding of deacylated tRNA acts as a switch, releasing the kinase domain from interacting with the HisRS-like domain, which is otherwise inhibitory for its kinase activity [45]. In support of these findings, mutations in the conserved residues of the HisRS-like domain required for tRNA binding (the m2 motif) impaired both tRNA binding and Gcn2 function [14,46]. In addition to tRNA binding by the HisRS-like domain, ribosome and tRNA binding by the CTD of Gcn2 are critical for its activity, whereby mutations of key lysine residues within this domain were found to inhibit eIF2α phosphorylation by the factor [14,45,47]. Collectively, these findings were key to establishing the 'well-accepted' model for Gcn2 activation by deacylated tRNAs on ribosomes.

### Gcn2 is activated under conditions that do not result in accumulation of deacylated tRNAs

Since its discovery, the majority of studies on Gcn2 took advantage of its robust activation in response to amino acid starvation. In yeast, for example, 3-aminotriazole (3-AT), an inhibitor of histidine biosynthesis, has been widely used to decipher the mechanism of Gcn2 activation [46,48,49]. Similarly, the addition of sulfometuron methyl, an inhibitor of isoleucine and valine biosynthesis [46,50], robustly activates Gcn2. As expected, the addition of these compounds results in the accumulation of deacylated tRNA^His^, tRNA^Ile^, and tRNA^Val^, respectively, which were proposed to be responsible for activating the kinase [51]. However, it was soon observed that general stress conditions that are not necessarily associated with the accumulation of deacylated tRNAs can also activate Gcn2. Among these are alkylating [52] and oxidizing agents, such as methyl methane sulfonate (MMS), and 4-nitroquinoline 1-oxide (4NQO) [9,53]. MMS alkylates RNA by modifying the oxygen and nitrogen atoms of nucleobases, resulting in the accumulation of adducts, such as m^1^A and m^3^C, while the UV-mimic 4NQO generates ROS and hence the oxidation of G, forming the 8-oxoG adduct. These modifications inhibit the tRNA selection process and result in widespread stalling [53–55]. As puzzling was the observation that in *S. pombe* and *S. cerevisiae*, the deletion of the tRNA-modifying gene *TRM7*, responsible for 2′-O-methylation of a subset of tRNAs at the anticodon loop, results in the activation of Gcn2 with no detectable decrease in tRNA aminoacylation [56]. In agreement with these observations, yeast lacking the tRNA-modifying machinery for adding 5-methoxycarbonylmethyl (mcm^5^) and 2-thio (s^2^) groups to uridine at the 5′ nucleotide of the anticodon of several tRNAs derepresses the translation of *GCN4* [57]. Interestingly, ribosome profiling showed slowed translation on the codons that are decoded by the hypomodified tRNAs. Although under these conditions, it was suggested that the ISR was induced in a Gcn2-independent mechanism.

The connection between ribosome slowdown and GCN2 activation is bolstered by observations that in mice neurons lacking a copy of a tRNA gene (tRNA^Arg^_UCU_) and the ribosome rescue factor GTPBP2, eIF2α was phosphorylated in a GCN2-dependent manner [58]. Similar to what was later observed in yeast, Ishimura and colleagues found no detectable reduction in tRNA aminoacylation. Instead, increased ribosome stalling was observed on the AGA codons, which are decoded by the hypomorphic tRNA. These findings suggested that mammalian GCN2, similar to the fungal one, can be activated in a deacylated-tRNA-independent manner, but is still dependent on ribosome binding. Indeed, two independent groups showed that the P stalk of the ribosome activates GCN2 much more efficiently than deacylated tRNA. In the first study, Inglis and colleagues went on to show that the protein protects components of the P stalk from hydrogen-deuterium exchange. They proposed a model by which the P stalk, and in particular the uL10 subunit, becomes available for GCN2 binding in the absence of eEF1A.aa-tRNA.GTP ternary complex, which binds the very same site on the ribosome during the elongation phase of translation [44]. In the second study, Harding and colleagues complemented and expanded on these findings by revealing, through CRISPR-Cas9 mutagenesis screen in Chinese hamster ovary (CHO) cells, that mutations in the P stalk proteins suppressed GCN2 activation. Ribosomes isolated from these mutants failed to stimulate GCN2-mediated phosphorylation of eIF2α, whereas those isolated from wildtype CHO cells stimulated the kinase activity [59]. As the P stalk is a critical region on the ribosome important for interacting with translation elongation factors, these findings imply that GCN2 is potently stimulated by ribosomes that have become stalled during translation. In agreement with these models, more recent studies showed that antibiotics that inhibit tRNA selection and stall ribosomes also activate Gcn2 in yeast cells [9,46]. Furthermore, the depletion of the release factor eRF1, which stalls ribosomes on stop codons, was found to induce eIF2α phosphorylation [46].

### Collided ribosomes signal for both translational quality control and the ISR

While these recent observations revealed a profound connection between ribosome stalling and activation of the ISR, the mechanism by which GCN2 and its co-activators distinguish between stalled ribosomes and elongating ones was not immediately understood. At the same time, several groups, including ours, were focused on studying the quality control processes associated with ribosome stalling. These mechanisms fulfill the important tasks of ribosome rescue and degradation of the defective mRNA and the incomplete nascent peptide. The details of these pathways are reviewed elsewhere [7,60–63]. However, relevant to our discussion here is that these pathways are activated by the same signals that trigger induction of the ISR. For instance, our group showed that mRNA damage, as induced by the alkylator MMS, for example, robustly activates ribosome quality control (RQC) [9,53,64]. As mentioned earlier, more than two decades ago, MMS was also shown to activate Gcn2-mediated phosphorylation of eIF2α [52].

At the time of the discovery that GCN2 can be activated by translational stress, the ribosome field was beginning to appreciate that the signal for initiating the RQC pathway is collided ribosomes. In particular, given the fast elongation rate of translation and the fact that multiple ribosomes often occupy the same mRNA molecule, a stalling event (if not resolved rapidly) would lead to a pileup of collided ribosomes. Indeed, the addition of compounds like MMS and 3-AT was observed to result in the accumulation of RNase-resistant disome and trisome structures, indicative of higher order ribosome structures [9,65–67]. We and others showed that these structures are responsible for recruiting and activating the E3 ligase Hel2 (ZNF598 in mammals), the key sensor for the RQC pathway [68–70]. Briefly, Hel2 adds K63-linked ubiquitin chains to several ribosomal proteins, which act as beacons for downstream effector molecules to recycle ribosomes and degrade the mRNA [68,70,71].

**Figure 3:**
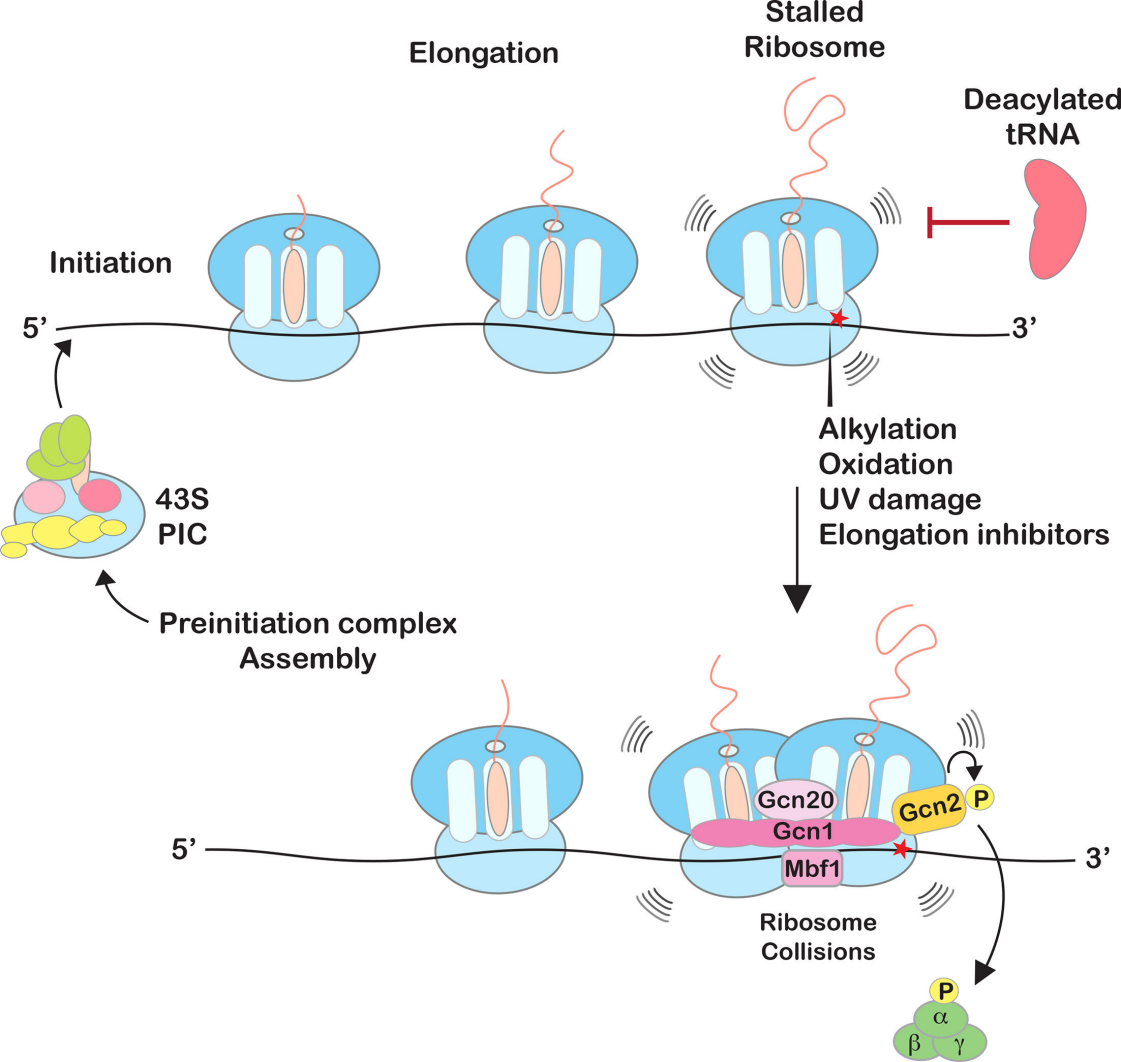
A model for general control nonderepressible 2 (Gcn2) activation on collided ribosomes. Elongating ribosomes stall on damaged mRNA, as well as when aminoacylation of tRNAs is inhibited in response to amino-acid starvation. Stalling eventually leads to ribosome collisions, which are recognized by Gcn2 and its co-activators (Gcn1, Gcn20, and Mbf1), activating the ISR through Gcn2-mediated phosphorylation of eIF2α.

Whether Gcn2 is activated through a similar mechanism was not known until Wu and colleagues made the discovery that collided ribosomes trigger the induction of several stress response pathways, including the ISR (Figure 3) and the ribotoxic stress response [66]. More specifically, they showed that in mammalian cells, GCN2 is activated in the presence of the antibiotic anisomycin, but only when it is added to intermediate concentrations that promote ribosome collisions. Similarly, UV damage was observed to induce phosphorylation of eIF2α, which was accompanied by increased ribosome collisions [66]. Highlighting the conservation of its activation, our group showed that in yeast, the addition of mRNA-damaging agents causes ribosome collisions and activates Gcn2 [9]. In support of these observations, two independent groups used cryo-EM analysis to reveal that Gcn2’s co-activator Gcn1 binds collided ribosomes, whereby its expansive HEAT repeats are used to extend its structure all the way from the colliding ribosome to the stalled one [40,72]. Notably, the two structures are of complexes that do not represent a Gcn2-activated state. Notwithstanding, there is evidence that Gcn2 activation requires an empty A site of the stalled ribosome, suggesting that the factor’s binding site on the ribosome overlaps with that of the incoming ternary complex [9,46]. Notably, in a recent Cryo-EM, Gcn2 was shown to exist as an inactive dimer in complex with the ribosomal 60S subunit, independent of its co-activators, indicative of a 'stand-by state'. However, in response to stress, a rapid shift of Gcn2 from the 60S subunit into the colliding ribosome occurred in the presence of its co-activators (Gcn1 or Gcn20). It was proposed that 60S binding by Gcn2 may provide a platform that enables relocalization to collided ribosomes for rapid activation of the ISR [36].

Emerging from many studies is that collided ribosomes activate multiple pathways, including RQC, the ISR, and RSR [7]. The choice of which process to activate over the others is paramount to cellular homeostasis. For example, the ISR and RQC processes lead to two completely distinct outcomes, and as a result, it is crucial that collided ribosomes activate the appropriate process. Highlighting the intricate co-ordination between these pathways is the observation that inactivation of one of these processes leads to overactivation of the other, suggesting that RQC and the ISR are in apparent competition with each other [9]. Furthermore, under conditions that cause low levels of stalling, RQC is robustly active, and only when stalling is more widespread is Gcn2 activated. More recently, our group provided molecular rationale for how the activities of the two processes are co-ordinated on collided ribosomes. Introducing active site mutants in Hel2, which display increased binding to collided ribosomes, enhances the activation of the ISR, suggesting that binding of Hel2 to ribosomes does not inhibit Gcn2 activation. Instead, the inability to rescue collided ribosomes, when the factor is inactive, leads to activation of Gcn2. Indeed, mutating the Hel2 target lysine residues in uS10, which also inhibits ribosome rescue, leads to overactivation of the ISR. Interestingly, RQC is limited by Hel2 levels, as the overexpression of the factor causes a dramatic reduction in the induction of Gcn4 targets, while increasing stalling-induced ribosomal protein ubiquitination [64]. Therefore, it appears that through evolution, organisms fine-tuned the levels and affinities of their RQC and ISR factors, ensuring that RQC is more robustly activated to rescue stalled ribosomes but is quickly exhausted. This, in turn, allows for Gcn2 activation only under severe stress conditions when Hel2 is overwhelmed by ribosome collisions. Recently, Coria and colleagues revealed a new role for the ISR in clearing defective ribosomes, which are subject to nonfunctional ribosome decay (NRD) [73]. In this model, NRD is dependent on the activation of GCN2 to limit translation initiation, which prevents collisions between the scanning small subunit and the defective ribosome stalled on the initiation codon [74].

### Mbf1 is a co-activator of Gcn2 on collided ribosomes

As stated earlier, reprogramming of gene expression in response to the induction of the ISR in yeast is largely transmitted through translational derepression of the key transcription factor Gcn4. Early studies on the transcriptional activity of Gcn4 suggested that it requires the co-activation function of the multiprotein bridging factor 1 (Mbf1) to recruit the basal transcription machinery to its targets [75,76]. In particular, it was proposed that Mbf1 bridges a connection between Gcn4 and TATA binding protein. Later studies in many eukaryotes showed the factor to be important for stress response. Remarkably, few studies in silkworm and yeast were used by the field to explain the role of the factor during transcriptional response to environmental changes in mammals and plants [77,78]. In contrast with this proposed role in mediating the activity of stress-induced transcription factors, the archaeal homologue of the factor was shown to associate with the small ribosomal subunit [79]. Similarly, proximity-labeling approaches suggested that yeast Mbf1 interacts with ribosomal proteins [80]. More recently, we and others showed that the factor and its human homologue bind collided ribosomes, during which it functions to maintain the reading frame [40,65,81]. These observations were puzzling because they suggest that Mbf1 is a translation-based factor in the cytoplasm [82], yet it has an important function in transcription in the nucleus.

The observation that collided ribosomes activate the ISR and recruit Mbf1 suggested an alternative mechanism for how this factor might function during induction of the ISR. Indeed, in a very recent study from our group, Mbf1 was found to be required for optimal Gcn2 activation, and in its absence, eIF2α phosphorylation and Gcn4 accumulation are dramatically reduced in response to all tested stress conditions. More importantly, by constitutively expressing GCN4 (bypassing the requirement of Gcn2 activation for GCN4 translation), we showed that Mbf1 has no transcriptional co-activation function. This newly described role of Mbf1 during Gcn2 activation is critically dependent on its ability to be recruited to collided ribosomes. Mutations in the factor or in the ribosomes that inhibit this recruitment profoundly reduce Gcn2 activation in the presence of stress [65]. As a result, we would argue that Mbf1 proteins should be reclassified as sensors of stress (like Gcn2’s co-activators Gcn1 and Gcn20) and not as mediators of transcription (Figure 3). Notably, these findings add vital support for the role of collided ribosomes in inducing one of the most important stress responses in eukaryotes.

## Conclusion

As detailed throughout this review, studies in the past few years have reshaped our understanding of the mechanism by which the ISR is activated by GCN2. While ribosome binding by the kinase and its co-activators has long been documented to be critical for the process, their role was assumed to be limited to acting as a platform for delivering deacylated tRNA to GCN2. Recent studies have upended this view, bringing ribosomes front and center during GCN2-mediated activation of the ISR. This mode of activation provides a more holistic approach, integrating multiple observations and studies on the ISR. In particular, the elongation phase of translation is exquisitely sensitive to a diverse set of stress conditions, beyond amino-acid deprivation; all of which have the capability of stalling the ribosome and causing them to collide [7] (Figure 3). This, in turn, must have necessitated the evolution of stress responses that can utilize the resulting structures of collided ribosomes as sensors, instead of depending on the accumulation of deacylated tRNAs, which is limited to a restricted set of stress conditions. Indeed, collided ribosomes have been recently recognized as molecular sentinels, incorporating stress signals into several important biological pathways beyond the ISR. For instance, collided ribosomes activate the RSR through ZAKα, which appears to be responsible for inducing apoptosis and activating the inflammasome in response to acute UV-mediated damage of RNA [4]. Interestingly, similar to what was observed for RQC-mediated suppression of the ISR through ribosome rescue, GCN2 activation suppresses the induction of RSR by limiting the concentration of collided ribosomes [4]. Emerging from all these studies is that the dosage and duration of stress are key to activating the appropriate response.

Notably, how collided ribosomes activate these various pathways is not completely understood. Although now we have the structure of inactive Gcn2 dimer bound to the 60S subunit [36], in the absence of a structure of the factor in complex with collided ribosomes (in the presence of its co-activators Gcn1, Gcn20, and Mbf1), it remains unclear how Gcn2 is specifically activated by stalled/collided ribosomes and the role of the P stalk and the A site during the process. The cryo-EM structures of Gcn1 and Mbf1 in complex with collided ribosomes offer some clues about the architecture of these higher order structures but provide no concrete insights into how these co-activators help Gcn2 carry out its function [40]. Intriguingly, GCN1 has multiple quality-control functions that include the recruitment of factors to degrade translation factors that are stuck on the ribosome, as well as proteins cross-linked onto the mRNA [72,83,84]. Furthermore, the factor appears to have a wide role during readthrough events and stalling on nonoptimal codons, during which it is proposed to recruit the mRNA degradation machinery [85]. All these roles depend on the ability of the factor to recognize collided ribosomes. How GCN1 is able to recruit a diverse set of factors to collided ribosomes, depending on the type of stall, is ambiguous. Intriguingly, two recent studies have argued for stalling-dependent and -independent modes of activation of GCN2, whereby the latter mechanism requires deacylated tRNAs for the induction [46,86]. Both pathways still require GCN1, and as a result, what role GCN1 plays during this collision-independent mechanism is also not understood.

Finally, in addition to the ISR, nutrient availability regulates a number of processes, including the target of rapamycin (TOR) pathway. For reviews on the TOR pathway, please refer to Refs. [87–92]. Briefly and similar to the ISR, TOR regulates the initiation phase of translation, namely mRNA activation by the eIF4F complex. During the process of mRNA activation, eIF4E of the eIF4F complex recognizes the cap structure of the mRNA and recruits the helicase eIF4A and the scaffold protein eIF4G [18]. Under starvation conditions, TOR is inhibited, leading to sequestration of eIF4E, through the dephosphorylation of eIF4E-BPs. This leads to the inhibition of cap-dependent translation [93]. Thus, the same conditions that activate GCN2 inhibit TOR, suggesting that their activities must be co-ordinated. Indeed, several studies have provided evidence for cross-talk between the two pathways. In two recent reports, our group and the Ingolia group showed that eIF4E depletion in yeast (mimicking the effect of TOR inhibition on mRNA activation) leads to the induction of the ISR, but notably in an eIF2α phosphorylation-independent manner [19,94]. We still lack a mechanistic understanding by which these two seemingly independent pathways intersect in response to stress, and it will be interesting to address these important questions.

**Perspectives**

- Gcn2-mediated activation of the integrated stress response (ISR) is a conserved eukaryotic signaling process that is critical for organisms to adapt to environmental changes that affects ribosome function. The activation of general control nonderepressible 2 (Gcn2) inhibits global protein synthesis through phosphorylation of eukaryotic initiation factor 2α, while promoting the translation of pro-survival mRNAs.
- Initial models suggested that Gcn2 is activated in response to amino acid deprivation through sensing deacylated tRNAs that accumulate on ribosomes. Recent studies have shown that translational stress conditions that stall the ribosome result in collisions, which are sensed by several quality control and stress response pathways, including the ISR through Gcn2 and its co-activators.
- The mechanism by which Gcn2 and its co-activators sense collided ribosomes to trigger the induction of the ISR is poorly understood. Equally important is the interplay between this pathway and other signaling processes that respond to translational stress and nutrient availability. The ISR is critical for maintaining cellular homeostasis in response to endogenous and exogenous agents that could compromise the translational machinery of the cell. As a result, its dysregulation has been associated with neurodegeneration and cancer. Recent efforts have focused on developing novel therapeutics that can target the ISR for potential neuroprotection.

## Open Access

This article has been published open access under our Subscribe to Open programme, made possible through the support of our subscribing institutions, learn more here: https://portlandpress.com/pages/open_access_options_and_prices#conditional

## Abbreviations

3-AT: 3-Aminotriazole
ATF4: Activating Transcription Factor 4
CHO: Chinese hamster ovary
CTD: C-terminal domain
GCN2: general control nonderepressible 2
GTPB2: GTP binding protein 2
HRI: Heme-Regulated Inhibitor
ISR: Integrated Stress Response
MMS: methyl methanesulfonate
MMS: Methyl Methane Sulfonate
Mbf1: Multiprotein bridging factor 1
4NQO: 4-Nitroquinoline 1-oxide
NRD: nonfunctional ribosome decay
NTD: N-terminal domain
PKR: Protein Kinase R
ROS: reactive oxygen species
RQC: ribosome quality control
RSR: Ribotoxic stress response
TOR: target of rapamycin
eEF3: eukaryotic elongation factor 3
eIF2: eukaryotic initiation factor 2
uORFs: upstream Open Reading Frames

## References

[1] Pakos-Zebrucka K., Koryga I., Mnich K., Ljujic M., Samali A., Gorman A.M (2016). The integrated stress response. EMBO Rep..

[2] Costa-Mattioli M., Walter P (2020). The integrated stress response: from mechanism to disease. Science.

[3] Hinnebusch A.G (2005). Translational regulation of GCN4 and the general amino acid control of yeast. Annu. Rev. Microbiol..

[4] Sinha N.K., McKenney C., Yeow Z.Y., Li J.J., Nam K.H., Yaron-Barir T.M. (2024). The ribotoxic stress response drives UV-mediated cell death. Cell.

[5] Harding H.P., Novoa I., Zhang Y., Zeng H., Wek R., Schapira M. (2000). Regulated translation initiation controls stress-induced gene expression in mammalian cells. Mol. Cell.

[6] Donnelly N., Gorman A.M., Gupta S., Samali A (2013). The eIF2α kinases: their structures and functions. Cell. Mol. Life Sci..

[7] Kim K.Q., Zaher H.S (2022). Canary in a coal mine: collided ribosomes as sensors of cellular conditions. Trends Biochem. Sci..

[8] Masson G.R (2019). Towards a model of GCN2 activation. Biochem. Soc. Trans..

[9] Yan L.L., Zaher H.S (2021). Ribosome quality control antagonizes the activation of the integrated stress response on colliding ribosomes. Mol Cell.

[10] Clemens M.J., Safer B., Merrick W.C., Anderson W.F., andLondon I.M (1975). Inhibition of protein synthesis in rabbit reticulocyte lysates by double-stranded RNA and oxidized glutathione: indirect mode of action on polypeptide chain initiation. Proc Natl Acad Sci USA.

[11] Fagard R., London I.M (1981). Relationship between phosphorylation and activity of heme-regulated eukaryotic initiation factor 2 alpha kinase. Proc. Natl. Acad. Sci. U.S.A..

[12] Levin D.H., Petryshyn R., London I.M (1980). Characterization of double-stranded-RNA-activated kinase that phosphorylates alpha subunit of eukaryotic initiation factor 2 (eIF-2 alpha) in reticulocyte lysates. Proc. Natl. Acad. Sci. U.S.A..

[13] Hinnebusch A.G., Fink G.R (1983). Positive regulation in the general amino acid control of Saccharomyces cerevisiae. Proc Natl Acad Sci USA.

[14] Wek S.A., Zhu S., Wek R.C (1995). The histidyl-tRNA synthetase-related sequence in the eIF-2 alpha protein kinase GCN2 interacts with tRNA and is required for activation in response to starvation for different amino acids. Mol. Cell. Biol..

[15] Hinnebusch A.G (1988). Mechanisms of gene regulation in the general control of amino acid biosynthesis in Saccharomyces cerevisiae. Microbiol. Rev..

[16] Chen J.J., Throop M.S., Gehrke L., Kuo I., Pal J.K., Brodsky M. (1991). Cloning of the cDNA of the heme-regulated eukaryotic initiation factor 2 alpha (eIF-2 alpha) kinase of rabbit reticulocytes: homology to yeast GCN2 protein kinase and human double-stranded-RNA-dependent eIF-2 alpha kinase. Proc. Natl. Acad. Sci. U.S.A..

[17] Dever T.E., Feng L., Wek R.C., Cigan A.M., Donahue T.F., Hinnebusch A.G (1992). Phosphorylation of initiation factor 2 alpha by protein kinase GCN2 mediates gene-specific translational control of GCN4 in yeast. Cell.

[18] Jackson R.J., Hellen C.U.T., Pestova T.V (2010). The mechanism of eukaryotic translation initiation and principles of its regulation. Nat. Rev. Mol. Cell Biol..

[19] Kim K.Q., Nanjaraj Urs A.N., Lasehinde V., Greenlaw A.C., Hudson B.H., Zaher H.S (2024). eIF4F complex dynamics are important for the activation of the integrated stress response. Mol. Cell.

[20] Sudhakar A., Ramachandran A., Ghosh S., Hasnain S.E., Kaufman R.J., Ramaiah K.V (2000). Phosphorylation of serine 51 in initiation factor 2 alpha (eIF2 alpha) promotes complex formation between eIF2 alpha(P) and eIF2B and causes inhibition in the guanine nucleotide exchange activity of eIF2B. Biochemistry.

[21] Krishnamoorthy T., Pavitt G.D., Zhang F., Dever T.E., Hinnebusch A.G (2001). Tight binding of the phosphorylated alpha subunit of initiation factor 2 (eIF2alpha) to the regulatory subunits of guanine nucleotide exchange factor eIF2B is required for inhibition of translation initiation. Mol. Cell. Biol..

[22] Gomez E., Mohammad S.S., Pavitt G.D (2002). Characterization of the minimal catalytic domain within eIF2B: the guanine-nucleotide exchange factor for translation initiation. EMBO J..

[23] Rowlands A.G., Panniers R., Henshaw E.C (1988). The catalytic mechanism of guanine nucleotide exchange factor action and competitive inhibition by phosphorylated eukaryotic initiation factor 2. J. Biol. Chem..

[24] B’chir W., Maurin A.C., Carraro V., Averous J., Jousse C., Muranishi Y. (2013). The eIF2α/ATF4 pathway is essential for stress-induced autophagy gene expression. Nucleic Acids Res..

[25] Kroemer G., Mariño G., Levine B (2010). Autophagy and the integrated stress response. Mol. Cell.

[26] Gulias J.F., Niesi F., Arán M., Correa-García S., Bermúdez-Moretti M (2023). Gcn4 impacts metabolic fluxes to promote yeast chronological lifespan. Plos One.

[27] Hinnebusch A.G., Natarajan K (2002). Gcn4p, a master regulator of gene expression, is controlled at multiple levels by diverse signals of starvation and stress. Eukaryotic Cell.

[28] Vattem K.M., Wek R.C (2004). Reinitiation involving upstream ORFs regulates ATF4 mRNA translation in mammalian cells. Proc. Natl. Acad. Sci. U.S.A..

[29] Dever T.E., Ivanov I.P., Hinnebusch A.G (2023). Translational regulation by uORFs and start codon selection stringency. Genes Dev..

[30] Hinnebusch A.G (1997). Translational regulation of yeast GCN4. A window on factors that control initiator-trna binding to the ribosome. J. Biol. Chem..

[31] Wek R.C., Jackson B.M., Hinnebusch A.G (1989). Juxtaposition of domains homologous to protein kinases and histidyl-tRNA synthetases in GCN2 protein suggests a mechanism for coupling GCN4 expression to amino acid availability. Proc. Natl. Acad. Sci. U.S.A..

[32] Wek R.C., Ramirez M., Jackson B.M., Hinnebusch A.G (1990). Identification of positive-acting domains in GCN2 protein kinase required for translational activation of GCN4 expression. Mol. Cell. Biol..

[33] Marton M.J., Vazquez de S., Aldana C.R., Qiu H., Chakraburtty K., andHinnebusch A.G (1997). Evidence that GCN1 and GCN20, translational regulators of GCN4, function on elongating ribosomes in activation of eIF2alpha kinase GCN2. Mol Cell Biol.

[34] Garcia-Barrio M., Dong J., Ufano S., Hinnebusch A.G (2000). Association of GCN1-GCN20 regulatory complex with the N-terminus of eIF2alpha kinase GCN2 is required for GCN2 activation. EMBO J..

[35] Qiu H., Garcia-Barrio M.T., Hinnebusch A.G (1998). Dimerization by translation initiation factor 2 kinase GCN2 is mediated by interactions in the C-terminal ribosome-binding region and the protein kinase domain. Mol. Cell. Biol..

[36] Paternoga H., Xia L., Dimitrova-Paternoga L., Li S., Yan L.L., Oestereich M. (2025). Structure of A Gcn2 dimer in complex with the large 60S ribosomal subunit. Proc Natl Acad Sci USA.

[37] Marton M.J., Crouch D., Hinnebusch A.G (1993). GCN1, a translational activator of GCN4 in Saccharomyces cerevisiae, is required for phosphorylation of eukaryotic translation initiation factor 2 by protein kinase GCN2. Mol. Cell. Biol..

[38] Sattlegger E., Hinnebusch A.G (2000). Separate domains in GCN1 for binding protein kinase GCN2 and ribosomes are required for GCN2 activation in amino acid-starved cells. EMBO J..

[39] Vazquez de Aldana C.R., Marton M.J., Hinnebusch A.G (1995). GCN20, a novel ATP binding cassette protein, and GCN1 reside in a complex that mediates activation of the eIF-2 alpha kinase GCN2 in amino acid-starved cells. EMBO J..

[40] Pochopien A.A., Beckert B., Kasvandik S., Berninghausen O., Beckmann R., Tenson T. (2021). Structure of Gcn1 bound to stalled and colliding 80S ribosomes. Proc. Natl. Acad. Sci. U.S.A..

[41] Atkinson G.C., Tenson T., Hauryliuk V (2011). The RelA/SpoT homolog (RSH) superfamily: distribution and functional evolution of ppGpp synthetases and hydrolases across the tree of life. Plos One.

[42] Brown A., Fernández I.S., Gordiyenko Y., Ramakrishnan V (2016). Ribosome-dependent activation of stringent control. Nature.

[43] Castilho B.A., Shanmugam R., Silva R.C., Ramesh R., Himme B.M., Sattlegger E (2014). Keeping the eIF2 alpha kinase Gcn2 in check. Biochim Biophys Acta.

[44] Inglis A.J., Masson G.R., Shao S., Perisic O., McLaughlin S.H., Hegde R.S. (2019). Activation of GCN2 by the ribosomal P-stalk. Proc Natl Acad Sci USA.

[45] Dong J., Qiu H., Garcia-Barrio M., Anderson J., Hinnebusch A.G (2000). Uncharged tRNA activates GCN2 by displacing the protein kinase moiety from a bipartite tRNA-binding domain. Mol. Cell.

[46] Gupta R., Hinnebusch A.G (2023). Differential requirements for P stalk components in activating yeast protein kinase Gcn2 by stalled ribosomes during stress. Proc. Natl. Acad. Sci. U.S.A..

[47] Zhu S., Wek R.C (1998). Ribosome-binding domain of eukaryotic initiation factor-2 kinase GCN2 facilitates translation control. J. Biol. Chem..

[48] Klopotowski T., Wiater A (1965). Synergism of aminotriazole and phosphate on the inhibition of yeast imidazole glycerol phosphate dehydratase. Arch. Biochem. Biophys..

[49] Albrecht G., Mösch H.U., Hoffmann B., Reusser U., Braus G.H (1998). Monitoring the Gcn4 protein-mediated response in the yeast *Saccharomyces cerevisiae*. J. Biol. Chem..

[50] LaRossa R.A., Schloss J.V (1984). The sulfonylurea herbicide sulfometuron methyl is an extremely potent and selective inhibitor of acetolactate synthase in *Salmonella typhimurium*. J. Biol. Chem..

[51] Lageix S., Zhang J., Rothenburg S., Hinnebusch A.G (2015). Interaction between the tRNA-binding and C-terminal domains of Yeast Gcn2 regulates kinase activity *in vivo*. Plos Genet.

[52] Natarajan K., Meyer M.R., Jackson B.M., Slade D., Roberts C., Hinnebusch A.G. (2001). Transcriptional profiling shows that Gcn4p is a master regulator of gene expression during amino acid starvation in yeast. Mol. Cell. Biol..

[53] Yan L.L., Simms C.L., McLoughlin F., Vierstra R.D., Zaher H.S (2019). Oxidation and alkylation stresses activate ribosome-quality control. Nat. Commun..

[54] Simms C.L., Hudson B.H., Mosior J.W., Rangwala A.S., Zaher H.S (2014). An active role for the ribosome in determining the fate of oxidized mRNA. Cell Rep..

[55] Thomas E.N., Kim K.Q., McHugh E.P., Marcinkiewicz T., Zaher H.S (2020). Alkylative damage of mRNA leads to ribosome stalling and rescue by *trans* translation in bacteria. Elife.

[56] Han L., Guy M.P., Kon Y., Phizicky E.M (2018). Lack of 2’-O-methylation in the tRNA anticodon loop of two phylogenetically distant yeast species activates the general amino acid control pathway. PLoS Genet..

[57] Zinshteyn B., Gilbert W.V (2013). Loss of a conserved tRNA anticodon modification perturbs cellular signaling. PLoS Genet..

[58] Ishimura R., Nagy G., Dotu I., Chuang J.H., Ackerman S.L (2016). Activation of GCN2 kinase by ribosome stalling links translation elongation with translation initiation. Elife.

[59] Harding H.P., Ordonez A., Allen F., Parts L., Inglis A.J., Williams R.L. (2019). The ribosomal P-stalk couples amino acid starvation to GCN2 activation in mammalian cells. Elife.

[60] Brandman O., Hegde R.S (2016). Ribosome-associated protein quality control. Nat. Struct. Mol. Biol..

[61] D’Orazio K.N., Green R (2021). Ribosome states signal RNA quality control. Mol. Cell.

[62] Inada T., Beckmann R (2024). Mechanisms of translation-coupled quality control. J. Mol. Biol..

[63] Yip M.C.J., Shao S (2021). Detecting and rescuing stalled ribosomes. Trends Biochem. Sci..

[64] Nanjaraj Urs A.N., Lasehinde V., Kim L., McDonald E., Yan L.L., Zaher H.S (2024). Inability to rescue stalled ribosomes results in overactivation of the integrated stress response. J. Biol. Chem..

[65] Kim K.Q., Li J.J., Nanjaraj Urs A.N., Pacheco M.E., Lasehinde V., Denk T. (2024). Multiprotein bridging factor 1 is required for robust activation of the integrated stress response on collided ribosomes. Mol. Cell.

[66] Wu C.C.C., Peterson A., Zinshteyn B., Regot S., Green R (2020). Ribosome collisions trigger general stress responses to regulate cell fate. Cell.

[67] Meydan S., Guydosh N.R (2020). Disome and trisome profiling reveal genome-wide targets of ribosome quality control. Mol. Cell.

[68] Ikeuchi K., Tesina P., Matsuo Y., Sugiyama T., Cheng J., Saeki Y. (2019). Collided ribosomes form a unique structural interface to induce Hel2-driven quality control pathways. EMBO J..

[69] Juszkiewicz S., Chandrasekaran V., Lin Z., Kraatz S., Ramakrishnan V., Hegde R.S (2018). ZNF598 is a quality control sensor of collided ribosomes. Mol. Cell.

[70] Simms C.L., Yan L.L., Zaher H.S (2017). Ribosome collision is critical for quality control during No-Go decay. Mol. Cell.

[71] Saito K., Horikawa W., Ito K (2015). Inhibiting K63 polyubiquitination abolishes no-go type stalled translation surveillance in *Saccharomyces cerevisiae*. Plos Genet.

[72] Oltion K., Carelli J.D., Yang T., See S.K., Wang H.Y., Kampmann M. (2023). An E3 ligase network engages GCN1 to promote the degradation of translation factors on stalled ribosomes. Cell.

[73] LaRiviere F.J., Cole S.E., Ferullo D.J., Moore M.J (2006). A late-acting quality control process for mature eukaryotic rRNAs. Mol. Cell.

[74] Coria A.R., Shah A., Shafieinouri M., Taylor S.J., Orgebin E., Guiblet W. (2025). The integrated stress response regulates 18S nonfunctional rRNA decay in mammals. Mol. Cell.

[75] Takemaru K. i, Li F.Q., Ueda H., Hirose S (1997). Multiprotein bridging factor 1 (MBF1) is an evolutionarily conserved transcriptional coactivator that connects a regulatory factor and TATA element-binding protein. Proc. Natl. Acad. Sci. U.S.A..

[76] Takemaru K., Harashima S., Ueda H., Hirose S (1998). Yeast coactivator MBF1 mediates GCN4-dependent transcriptional activation. Mol. Cell. Biol..

[77] Jaimes-Miranda F., Chávez Montes R.A (2020). The plant MBF1 protein family: a bridge between stress and transcription. J. Exp. Bot..

[78] Kabe Y., Goto M., Shima D., Imai T., Wada T., Morohashi K. i (1999). The role of human MBF1 as a transcriptional coactivator. J. Biol. Chem..

[79] Blombach F., Launay H., Snijders A.P.L., Zorraquino V., Wu H., de Koning B (2014). Archaeal MBF1 binds to 30S and 70S ribosomes via its helix-turn-helix domain. Biochem. J..

[80] Opitz N., Schmitt K., Hofer-Pretz V., Neumann B., Krebber H., Braus G.H. (2017). Capturing the Asc1p/receptor for activated C kinase 1 (RACK1) microenvironment at the head region of the 40S ribosome with quantitative BioID in yeast. Mol Cell Proteomics.

[81] Sinha N.K., Ordureau A., Best K., Saba J.A., Zinshteyn B., Sundaramoorthy E. (2020). EDF1 coordinates cellular responses to ribosome collisions. Elife.

[82] Wang J., Zhou J., Yang Q., Grayhack E.J (2018). Multi-protein bridging factor 1(Mbf1), Rps3 and Asc1 prevent stalled ribosomes from frameshifting. Elife.

[83] Ford P.W., Bennett E.J (2023). How degrading! trapped translation factors get trashed. Cell Rep.

[84] Gurzeler L.A., Link M., Ibig Y., Schmidt I., Galuba O., Schoenbett J. (2023). Drug-induced eRF1 degradation promotes readthrough and reveals a new branch of ribosome quality control. Cell Rep..

[85] Houston L., Platten E.M., Connelly S.M., Wang J., Grayhack E.J (2022). Frameshifting at collided ribosomes is modulated by elongation factor eEF3 and by integrated stress response regulators Gcn1 and Gcn20. RNA.

[86] Misra J., Carlson K.R., Spandau D.F., Wek R.C (2024). Multiple mechanisms activate GCN2 eIF2 kinase in response to diverse stress conditions. Nucleic Acids Res..

[87] Wullschleger S., Loewith R., Hall M.N (2006). TOR signaling in growth and metabolism. Cell.

[88] González A., Hall M.N (2017). Nutrient sensing and TOR signaling in yeast and mammals. EMBO J..

[89] Loewith R., Hall M.N (2011). Target of rapamycin (TOR) in nutrient signaling and growth control. Genetics.

[90] Laplante M., Sabatini D.M (2012). mTOR signaling in growth control and disease. Cell.

[91] Shimobayashi M., Hall M.N (2014). Making new contacts: the mTOR network in metabolism and signalling crosstalk. Nat. Rev. Mol. Cell Biol..

[92] Howell J.J., Ricoult S.J.H., Ben-Sahra I., Manning B.D (2013). A growing role for mTOR in promoting anabolic metabolism. Biochem. Soc. Trans..

[93] Gingras A.C., Raught B., Gygi S.P., Niedzwiecka A., Miron M., Burley S.K. (2001). Hierarchical phosphorylation of the translation inhibitor 4E-BP1. Genes Dev..

[94] Diamond P.D., McGlincy N.J., Ingolia N.T (2024). Depletion of cap-binding protein eIF4E dysregulates amino acid metabolic gene expression. Mol. Cell.

